# Analysis of the relationship between serum LAMC2, TROAP and clinical stages and prognosis of non-muscle invasive bladder cancer

**DOI:** 10.1515/med-2026-1481

**Published:** 2026-07-06

**Authors:** Xiaolong He, Haiqin Zhu, Yongming Huang, Xiaolin Deng, Jianrong Huang

**Affiliations:** Department of Urology, The Affiliated Ganzhou Hospital, Jiangxi Medical College, Nanchang University, 341000, Ganzhou City, China; Department of Personnel, The Affiliated Ganzhou Hospital, Jiangxi Medical College, Nanchang University, 341000, Ganzhou City, China

**Keywords:** bladder cancer, Laminin γ2, trophinin-associated protein, clinical stage, prognosis

## Abstract

**Objectives:**

To investigate the relationship between serum LAMC2 and TROAP levels and clinical stage and prognosis in non-muscle invasive bladder cancer (NMIBC) patients.

**Methods:**

Serum LAMC2 and TROAP levels were measured in 102 NMIBC patients and 104 healthy controls. NMIBC patients were followed up and grouped by survival status. Correlations, prognostic factors, and diagnostic value were analyzed.

**Results:**

The serum levels of LAMC2 and TROAP in the research group were higher than those in the control group, and the two were positively correlated (r=0.303, p=0.002). The proportion of patients with high serum expression of LAMC2 and TROAP in stages III-IV was higher than that in patients with low expression (p<0.05). The 3-year survival rate of patients with high expression of LAMC2 and TROAP was lower than that of patients with low expression of both (p<0.05). Elevated levels of serum LAMC2 and TROAP, as well as TNM stages III–IV, were risk factors for mortality in NMIBC patients (p<0.05). The combined diagnosis of serum LAMC2 and TROAP showed better AUC than single diagnosis (Z=2.234, 2.864, p<0.05).

**Conclusions:**

Elevated serum LAMC2 and TROAP are linked to advanced stage and poor prognosis in NMIBC. Their combination improves prognostic diagnostic value.

## Introduction

Bladder cancer (BC) is one of the most common malignant tumors in the urinary system, imposing a heavy burden on society annually, with over 200,000 deaths worldwide each year due to this disease [1]. BC is associated with multiple risk factors, such as smoking, chronic infection or irritation, and occupational exposure to polycyclic aromatic hydrocarbons, benzene, aromatic amines, and other carcinogenic chemicals [2]. In clinical practice, the gold standard examinations for bladder cancer also present certain limitations. Urinary cytology is limited by low sensitivity and is prone to misdiagnosis or missed diagnosis, whereas cystoscopy, as an invasive procedure, has low patient acceptance and carries a risk of urinary tract infection [3]. Therefore, there is an urgent need to find reliable non-invasive biomarkers for the early diagnosis of BC.

Increasing evidence suggests that Laminin *γ*2 (LAMC2) monomer, an important component of the epithelial basement membrane, plays a pathological role in various cancers, including pancreatic cancer and lung cancer [4], 5]. Previous studies have indicated that high levels of LAMC2 expression can promote the invasion, migration, and metastasis of human cancer cells, thereby affecting tumor tissue metastasis and recurrence [6], 7]. Additionally, some researchers have identified LAMC2 as a key gene affecting bladder cancer (BC) prognosis through integrated bioinformatics analyses [8]. Therefore, it is hypothesized that LAMC2 is closely associated with BC progression and patient outcomes.

Trophinin-associated protein (TROAP) is a cytoplasmic protein required for microtubule cytoskeleton regulation and spindle assembly, and its expression plays a key role in the occurrence and development of various types of cancer [9]. Relevant studies have shown that regulating the expression of TROAP can influence cancer cell proliferation and migration, thereby inhibiting the occurrence and progression of tumors such as endometrial cancer and esophageal cancer [10], [11], [12]. Other studies have also found that TROAP can affect the malignant biological behavior of urinary system cancer cells, such as renal cell carcinoma [13]. However, there is currently a lack of research on the relationship between serum LAMC2 and TROAP levels and the clinical staging and prognosis of NMIBC patients. Therefore, this study aims to detect serum LAMC2 and TROAP levels in NMIBC patients and analyze the correlation between their changes in levels and the patients’ clinical staging and prognosis, hoping to provide a reference for improving patient outcomes.

## Objects and methods

### Study subjects

The study subjects selected were 102 NMIBC patients (study group) admitted to our hospital from July 2018 to January 2022. Concurrently, 104 healthy individuals undergoing health check-ups at our hospital during the same period and of similar age were selected as the control group. Among them, the study group included 56 male and 46 female patients, aged 38–78 years, with an average age of (60.48 ± 9.13) years. The control group included 58 male and 46 female individuals, aged 41–80 years, with an average age of (61.19 ± 9.27) years. After comparative analysis of basic information such as age and sex between the two groups, the results showed no significant differences between the groups, and the two groups were comparable (p>0.05) (p>0.05). This study involving human participants was approved by the Medical Ethics Committee of The Affiliated Ganzhou Hospital, Jiangxi Medical College (approval No. GZSRMYY2018050005; approval date: 14 May 2018). The study was conducted in accordance with the Declaration of Helsinki, as revised in 2013. Written informed consent was obtained from all individuals included in this study or their legal guardians/legal representatives.

**Figure 1:**
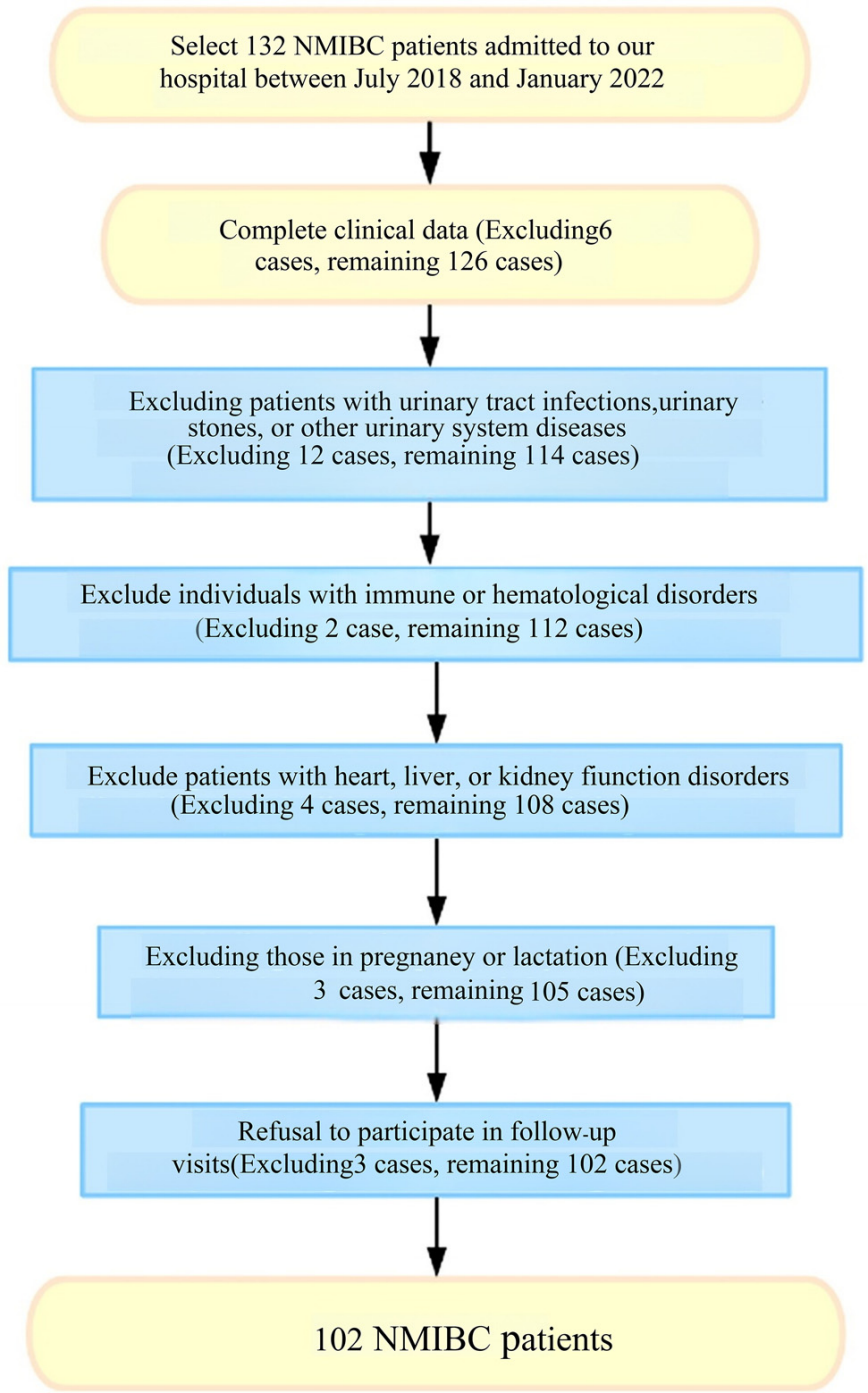
Case collection flowchart.

Inclusion criteria: ① NMIBC diagnosis met relevant diagnostic criteria [14], and all patients were confirmed by histological examination; ② Age >18 years, with complete clinical data; ③ Newly diagnosed; ④ Had not undergone surgery, radiotherapy, or chemotherapy before the operation. Exclusion criteria: ① Patients with concomitant urinary system diseases such as urinary tract infections or urolithiasis; ② Patients with concomitant immune or hematological diseases; ③ Patients who refused to participate in follow-up; ④ Patients with severe impairment of heart, liver, or kidney function; ⑤ Patients during pregnancy or lactation (see Figure 1).

### Methods

#### General data collection

General data of all subjects were collected, including: age, sex, body mass index (BMI), smoking history, lymph node metastasis, TNM stages, tumor diameter, depth of invasion, degree of differentiation, pathological grade.

#### Treatment

After admission, all patients underwent transurethral resection of bladder tumor (TURBT), followed by intravesical instillation of pirarubicin within 24 h postoperatively, with an instillation duration of 30 min. Postoperatively, intravesical instillation therapy was administered once weekly using the same drug and dosage as the immediate postoperative instillation, with the instillation time extended to 1 h, for a total of 8 weeks. Thereafter, the regimen was adjusted to once monthly instillation, continued for 1 year.

#### Detection of serum LAMC2 and TROAP levels

5 mL of venous blood was collected from patients on the day after admission and from healthy individuals on the day of their physical examination, under fasting conditions. The blood was centrifuged at 3,000 r/min for 10 min. The supernatant was separated, collected, and then stored at −20 °C for subsequent experiments. Serum LAMC2 and TROAP levels were detected by ELISA according to the manufacturers’ instructions. Materials used: LAMC2 (catalog number: ab282301) and TROAP were purchased from Abcam (USA) and Wuhan EIAab Science Co., Ltd., respectively. Absorbance was measured using a Multiskan FC microplate reader (Thermo Fisher Scientific, China).

#### Prognostic follow-up

All selected patients were systematically followed up for 3 years by telephone or outpatient visits after discharge. During the follow-up period, the survival status of the patients was observed and recorded. Follow-up was conducted every 3 months within 2 years postoperatively, and then every 6 months after 2 years. The follow-up ended by January 2025 or upon patient death. All patients completed the follow-up. Based on the follow-up results, the study group were divided into a survival group (64 cases) and a death group (38 cases).

### Statistical processing

SPSS 22.0 software was used for statistical analysis in this study. Count data were described using frequencies and/or percentages, and differences between groups were assessed using the chi-square test. Measurement data were expressed as mean ± standard deviation (SD), and comparisons between two groups were analyzed using the independent samples t-test. The Kaplan–Meier method was used to analyze the relationship between serum LAMC2 and TROAP levels and the prognosis of NMIBC patients; Risk factors for the prognosis of NMIBC patients were analyzed using multivariate Cox regression analysis; ROC curve analysis was used to assess the diagnostic value of serum LAMC2 and TROAP levels for the prognosis of NMIBC patients. A p-value <0.05 was considered statistically significant.

**Research ethics:** This study involving human participants was approved by the Medical Ethics Committee of The Affiliated Ganzhou Hospital, Jiangxi Medical College (approval No. GZSRMYY2018050005; approval date: 14-May-2018). The study was conducted in accordance with the Declaration of Helsinki, as revised in 2013.

**Informed consent:** Written informed consent was obtained from all individuals included in this study or their legal guardians/legal representatives.

## Results

### Comparison of serum LAMC2 and TROAP levels between the study group and the control group

As shown in Table 1, compared with the control group, the serum LAMC2 and TROAP levels in the study group patients were significantly elevated (p<0.05).

**Table 1:** Comparison of serum LAMC2 and TROAP levels between study group and control group patients.

Group	n	LAMC2, pg/mL	TROAP, ng/mL
Control group	104	51.47 ± 5.21	3.89 ± 0.39
Study group	102	89.47 ± 9.04	8.91 ± 0.91
*t*	–	37.052	51.632
p	–	<0.001	<0.001

### Correlation analysis of serum LAMC2 and TROAP levels

**Figure 2:**
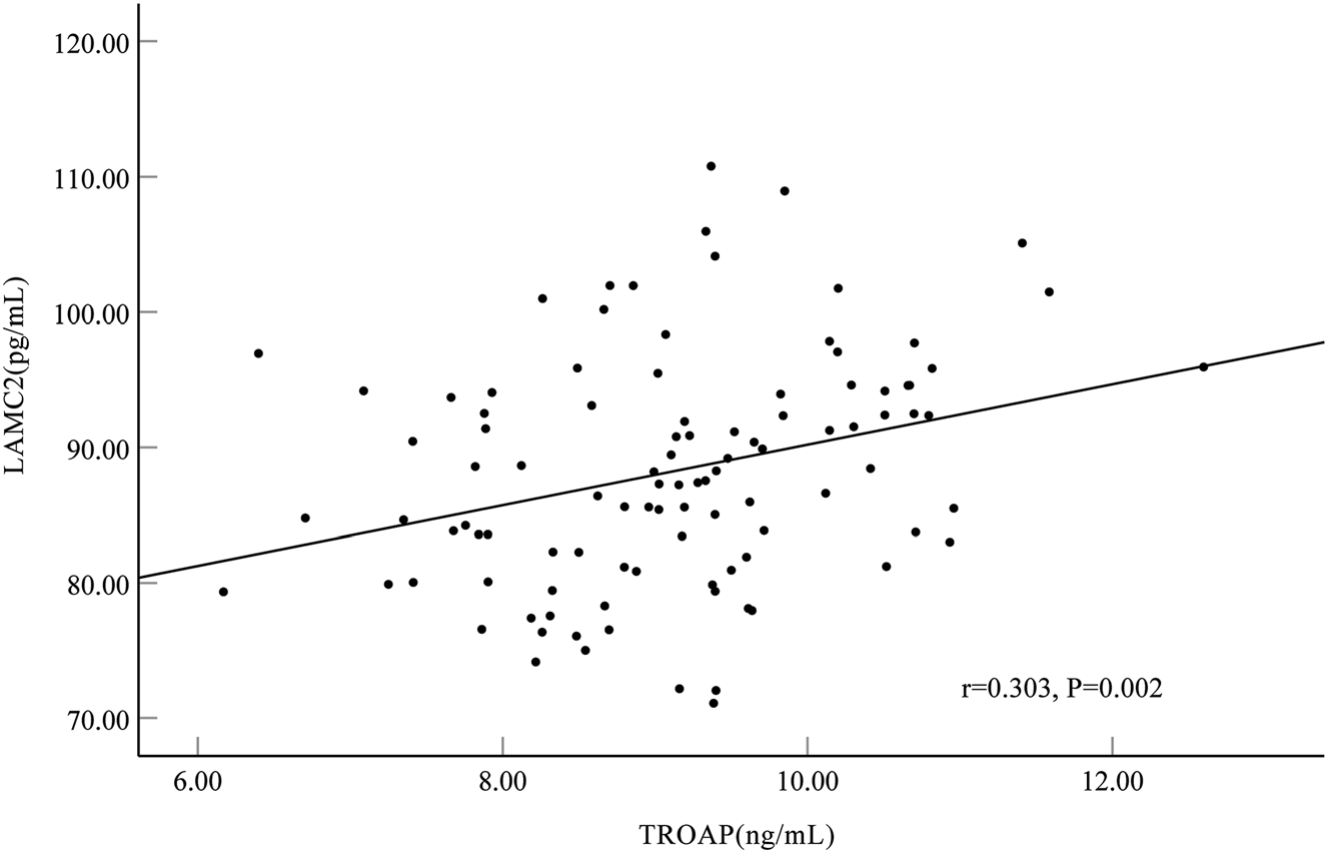
Correlation analysis of serum LAMC2 and TROAP levels.

The results of the correlation analysis showed that serum LAMC2 and TROAP levels were positively correlated (r=0.303, p=0.002) (see Figure 2).

### Relationship between serum LAMC2, TROAP levels and clinicopathological features of NMIBC patients

Using the mean values of serum LAMC2 and TROAP expression levels in the study group patients as cutoff values, they were divided into low expression and high expression groups. Serum LAMC2 and TROAP levels were not related to patient age, sex, BMI, smoking history, lymph node metastasis, tumor diameter, depth of invasion, degree of differentiation, or tumor pathological grade (p>0.05); they were related to patient TNM stages (p<0.05). In patients with TNM stages III–IV, the proportion of patients with high expression of serum LAMC2 and TROAP was higher than that of patients with low expression of LAMC2 and TROAP (p<0.05). The results are shown in Table 2.

**Table 2:** Relationship between serum LAMC2, TROAP levels and clinical pathological characteristics of BC patients.

Group	Age		Gender		BMI, kg/m^2^		Smoking history	
	<60	≥60	Male	Female	<24	≥24	Have	Not have
LAMC2 high expression (n=55)	27 (49.09)	28 (50.91)	31 (56.36)	24 (43.64)	20 (36.36)	35 (63.64)	29 (52.73)	26 (47.27)
LAMC2 low expression (n=47)	20 (42.55)	27 (57.45)	25 (53.19)	22 (46.81)	22 (46.81)	25 (53.19)	21 (44.68)	26 (55.32)
*χ* ^*2*^	0.436		0.103		1.141		0.657	
p	0.509		0.748		0.285		0.418	
TROAP high expression (n=54)	25 (46.30)	29 (53.70)	32 (59.26)	22 (40.74)	22 (40.74)	32 (59.26)	28 (51.85)	26 (48.15)
TROAP low expression (n=48)	22 (45.83)	26 (54.17)	24 (50.00)	24 (50.00)	25 (52.08)	23 (47.92)	21 (43.75)	27 (56.25)
*χ* ^*2*^	0.023		0.88		1.316		0.668	
p	0.879		0.348		0.251		0.414	

Group	Tumor diameter, cm		Lymph node metastasis	
	<3	≥3	Have	Not have
LAMC2 high expression (n=55)	34 (61.82)	21 (38.18)	34 (61.82)	21 (38.18)
LAMC2 low expression (n=47)	31 (65.96)	16 (34.04)	23 (48.94)	24 (51.06)
*χ* ^*2*^	0.188		1.706	
p	0.665		0.192	
TROAP high expression (n=54)	36 (66.67)	18 (33.33)	31 (57.41)	23 (42.59)
TROAP low expression (n=48)	29 (60.42)	19 (39.58)	26 (54.17)	22 (45.83)
*χ* ^*2*^	0.429		0.108	
p	0.512		0.742	

Group	TNM stages		Invasion depth		Degree of differentiation		Poorly differentiated	
	I∼II	IIIIV	Superficial	Deep	Medium/high	Differentiation	Tumor pathological grading	Low-level
LAMC2 high expression (n=55)	18 (32.73)	37 (67.27)	24 (43.64)	31 (56.36)	36 (65.45)	19 (34.55)	37 (67.27)	18 (32.73)
LAMC2 low expression (n=47)	31 (65.96)	16 (34.04)	19 (40.43)	28 (59.57)	28 (59.57)	19 (40.43)	27 (57.45)	20 (42.55)
*χ* ^*2*^	11.211		0.107		0.375		1.047	
p	0.001		0.743		0.54		0.306	
TROAP high expression (n=54)	17 (31.48)	37 (68.52)	26 (48.15)	28 (51.85)	37 (68.52)	17 (31.48)	33 (61.11)	21 (48.89)
TROAP low expression (n=48)	32 (66.67)	16 (33.33)	17 (35.42)	31 (64.58)	27 (56.25)	21 (43.75)	31 (64.58)	17 (35.42)
*χ* ^*2*^	12.603		1.689		1.636		0.131	
p	<0.001		0.194		0.201		0.717	

### Relationship between serum LAMC2, TROAP levels and prognosis of NMIBC patients

**Figure 3:**
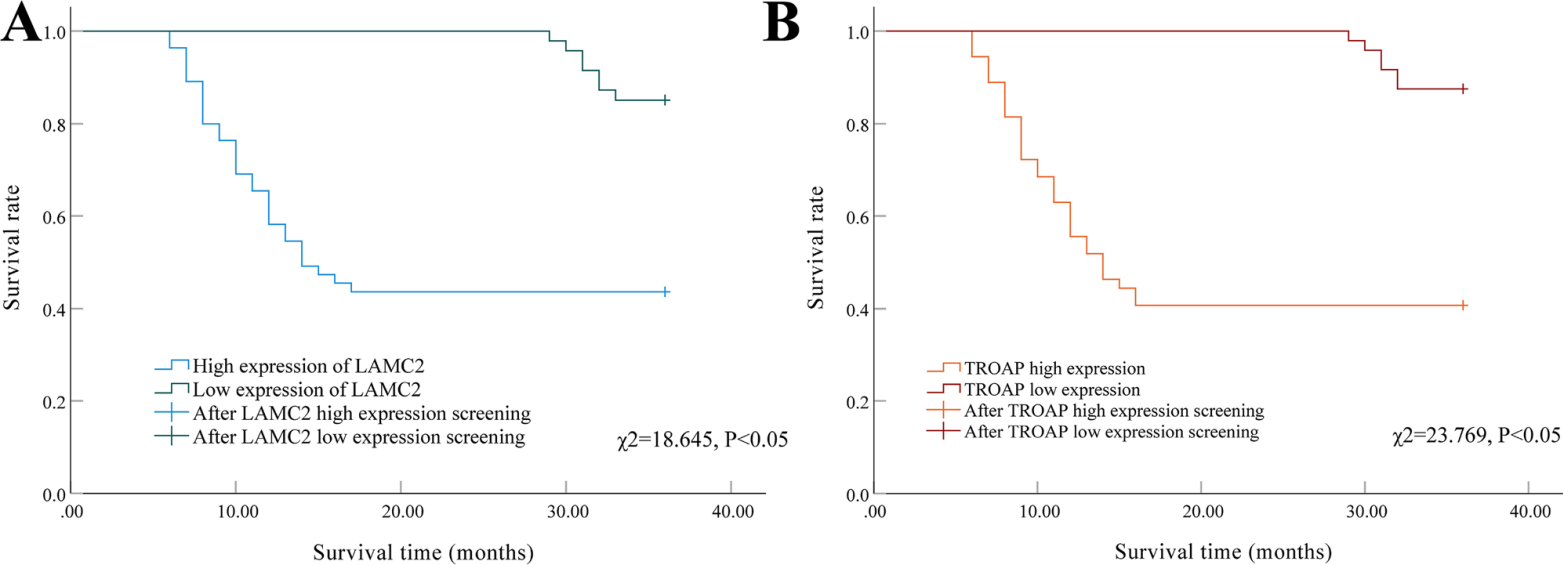
The relationship between serum LAMC2 and TROAP levels and the prognosis of NMIBC patients. A: Relationship between serum LAMC2 levels and 3-year survival rate of NMIBC patients; B: Relationship between serum TROAP levels and 3-year survival rate of NMIBC patients.

Survival curve results showed that the 3-year survival rate of patients with high serum LAMC2 expression (24/55, 43.64 %) was lower than that of patients with low LAMC2 expression (40/47, 85.11 %) (*χ*2=18.645, p<0.05); the 3-year survival rate of patients with high serum TROAP expression (22/54, 40.74 %) was lower than that of patients with low TROAP expression (42/48, 87.50 %) (*χ*2=23.769, p<0.05). The results are shown in Figure 3.

### Univariate analysis of prognostic death in NMIBC patients

As shown in Table 3, there were no significant differences in age, sex, lymph node metastasis, tumor diameter, depth of invasion, degree of differentiation, or tumor pathological grade between the survival group and the death group (p>0.05); compared to the survival group, the proportion of patients with TNM stages III–IV and serum LAMC2 and TROAP levels was significantly higher in the death group (p<0.05).

**Table 3:** Univariate analysis of prognostic mortality in BC patients.

Group	Age		Gender		Tumor diameter, cm		Lymph node metastasis	
	<60	≥60	Male	Female	<3	≥3	Have	Not have
Survival group (n=64)	26 (40.63)	38 (59.37)	36 (56.25)	28 (43.75)	39 (60.94)	25 (39.06)	36 (56.25)	28 (43.75)
Death group (n=38)	21 (55.26)	17 (44.74)	20 (59.09)	18 (40.91)	26 (68.42)	12 (31.58)	21 (55.26)	17 (44.74)
*χ* ^*2*^ */t*	2.056		0.086		0.578		0.012	
p	0.152		0.769		0.447		0.913	

Group	TNM stage		Invasion depth		Degree of differentiation	
	I∼II	III∼IV	Superficial	Deep	Medium/high	Differentiation
Survival group (n=64)	42 (65.63)	22 (34.37)	26 (40.63)	38 (59.37)	36 (52.94)	28 (47.06)
Death group (n=38)	7 (18.42)	31 (81.58)	17 (44.74)	21 (55.26)	28 (61.54)	10 (38.46)
*χ* ^*2*^ */t*	21.284		0.165		3.1	
p	<0.001		0.684		0.078	

Group	Poorly differentiated		LAMC2, pg/mL	TROAP, ng/mL
	Tumor pathological grading	Low-level		
Survival group (n=64)	39 (60.94)	25 (39.06)	85.01 ± 8.52	8.46 ± 0.86
Death group (n=38)	25 (65.79)	13 (34.21)	96.97 ± 9.72	9.66 ± 0.97
*χ* ^*2*^ */t*	0.24		6.501	6.494
p	0.624		<0.001	<0.001

### Analysis of factors affecting prognostic death in NMIBC patients

Taking the prognostic survival status of NMIBC patients as the dependent variable (death=1, survival=0), variables that showed statistically significant differences in the univariate analysis (p<0.05), including TNM stage (stage I–II=0, stage III–IV=1), LAMC2 (continuous variable), and TROAP (continuous variable), were included as independent variables in a multivariate Cox regression analysis using the forward stepwise method, and the final model was determined by minimizing the Akaike information criterion (AIC). The results showed that elevated serum LAMC2 and TROAP levels, and TNM stages III-IV were independent risk factors for death in NMIBC patients (p<0.05), see Table 4.

**Table 4:** Multivariate cox regression analysis of factors influencing prognostic death in BC patients.

Independent variable	β	SE	Wald	p-Value	HR	95 % CI
LAMC2	0.935	0.262	12.733	<0.001	2.547	1.524–4.256
TROAP	0.760	0.227	11.219	<0.001	2.139	1.371–3.338
TNM stage	1.389	0.421	10.890	<0.001	4.012	1.758–9.156

### Diagnostic value of serum LAMC2 and TROAP levels for prognostic death in NMIBC patients

**Figure 4:**
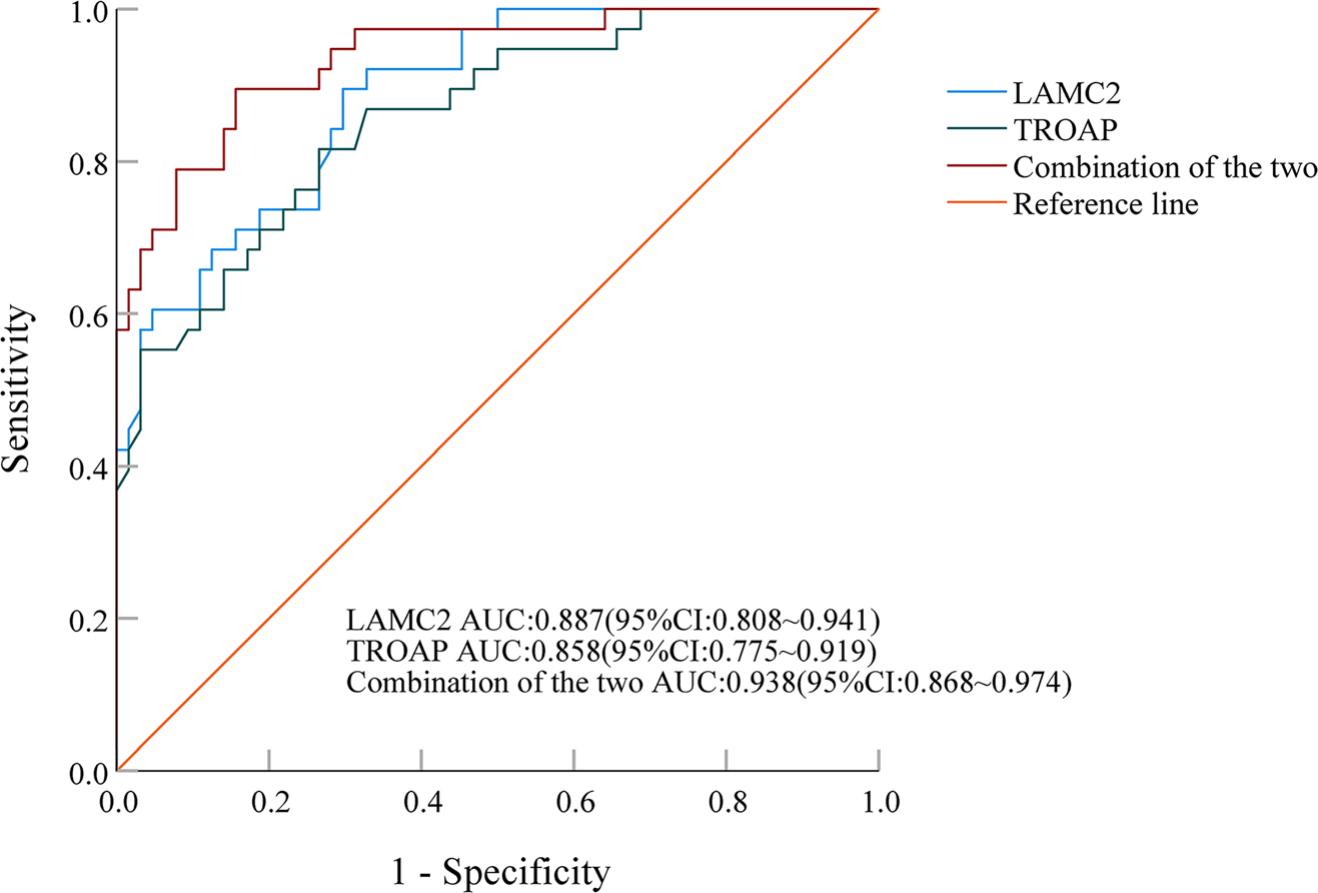
ROC curve of serum LAMC2 and TROAP levels for diagnosing prognosis and mortality in NMIBC patients.

ROC curves were plotted to analyze the diagnostic value of serum LAMC2 and TROAP levels for death in NMIBC patients. The results showed that the AUCs for serum LAMC2 and TROAP levels, individually and combined, in diagnosing prognostic death in NMIBC patients were 0.887, 0.858, and 0.938, respectively; the AUC of the combined diagnosis was superior to that of individual diagnoses (Z=2.234, 2.864, p<0.05). The results are shown in Figure 4, Table 5.

**Table 5:** ROC results of serum LAMC2 and TROAP levels for diagnosing prognosis and mortality in BC patients.

Index	Sensitivity (%)	Specificity (%)	Truncated value	AUC	95 % CI	Youden index
LAMC2	87.67	74.61	88.2 pg/mL	0.887	0.808–0.941	0.598
TROAP	81.58	73.44	8.99 ng/mL	0.858	0.775–0.919	0.550
Combination of the two	92.57	87.69	–	0.938	0.868–0.974	0.739

## Discussion

BC is an extremely common malignant tumor of the urogenital system. Approximately 70–80 % of patients are diagnosed with non-muscle-invasive tumors at the time of diagnosis, and these tumors can be effectively treated by transurethral resection [15]. Despite this, 20–30 % of patients at initial diagnosis have muscle-invasive BC, and approximately 30 % of NMIBC will further progress to muscle-invasive tumors [16]. For patients with muscle-invasive BC, radical cystectomy combined with extended pelvic lymph node dissection is a common treatment plan; even after undergoing such radical surgery, the long-term survival prospects for these patients remain poor, with a 5-year survival rate of approximately 50 % [17]. In recent years, the diagnosis, treatment, and prognosis of bladder cancer have not achieved satisfactory progress; similar to other malignant tumors, early diagnosis can avoid missing the surgical window or reduce adverse complications after radical cystectomy, thereby significantly improving the survival rate and quality of life for BC patients [18]. In BC research, some biomarkers have been identified that may aid in early diagnosis; however, these markers suffer from issues such as low accuracy or high invasiveness, failing to adequately meet the needs for early BC screening [19]. Therefore, exploring serum biomarkers with higher efficacy is of great significance for enhancing the early diagnosis accuracy of BC and improving prognosis.

LAMC2 is a key component of the heterotrimeric glycoprotein laminin-332 (LAM-332), which can regulate cell adhesion, differentiation, and migration, as well as the invasive behavior of epithelial cells in normal tissues [20]. Previous studies have found that LAMC2 is related to the invasion and metastasis of various cancer types, including colorectal cancer, pancreatic cancer, lung cancer, cervical cancer, and liver cancer [21]. Research by Liu et al. [22] demonstrated that LAMC2 promotes macrophage infiltration and extracellular matrix remodeling in non-small cell lung cancer (NSCLC), and LAMC2 plays an oncogenic role in the progression of NSCLC. Studies by Fu et al. [23] confirmed that matrix metalloproteinases (MMP) cleave and release domain III (DIII) of LAMC2 through proteolysis, and the released DIII fragment binds to epidermal growth factor receptors (EGFR), accelerating the proliferation, migration, and invasion of BC cells. The results of this study found that serum LAMC2 levels were elevated in BC patients, associated with the patients’ clinical stage and prognosis, and were an independent risk factor for poor prognosis in BC patients. Yang et al. [8], through integrated bioinformatics analyses, identified LAMC2 as a key gene affecting BC prognosis. Zheng et al. [24] found that upstream target genes can induce LAMC2 expression by promoting histone acetylation, thereby significantly facilitating bladder cancer progression. The underlying reason may be that LAMC2 undergoes proteolysis by MMPs, leading to the cleavage and release of the DIII fragment. This DIII fragment then binds to and interacts with EGFR, further promoting the proliferation, migration, and invasion of BC cells, with its overexpression playing an oncogenic role in the development of BC.

TROAP is considered crucial for cell proliferation, as it is necessary for spindle assembly and centrosome integrity during mitosis; meanwhile, TROAP is abnormally expressed in some tumor tissues and cell lines and can regulate the biological processes of cancer cells [25]. Previous studies have found that TROAP is highly expressed in some normal tissues and tumors, and high TROAP expression may indicate a poor prognosis or tumor progression in ovarian cancer, breast cancer, and hepatocellular carcinoma [26]. Research by Ye et al. [27] demonstrated that TROAP is highly expressed in prostate cancer (PCa) cells and is associated with shorter overall survival in PCa patients; TROAP may serve as a new prognostic biomarker to guide therapeutic intervention in PCa. Studies by Jin et al. [28] confirmed that TROAP promotes the proliferation, migration, and epithelial-mesenchymal transition (EMT) of PCa cells, while inhibiting apoptosis, by regulating the expression of transcription factors TWIST and c-Myc, thereby modulating S-phase progression of the cell cycle, and thus further promoting the occurrence and development of PCa. Chen et al. [29] identified TROAP as a candidate hub gene in BC using the CytoHubba plugin of Cytoscape. Jiang et al. [30], through bioinformatics analyses, determined TROAP as a potential prognostic hub gene in BC. Therefore, this study also measured and analyzed TROAP levels in NMIBC patients. The results of this study showed that serum TROAP expression was upregulated in NMIBC patients, related to the patients’ clinical stage and prognosis, and its elevated level was an independent risk factor for poor prognosis in NMIBC patients. Based on previous research findings, it is speculated that TROAP promotes the proliferation, migration, and EMT of BC cells and inhibits apoptosis by regulating the expression of relevant transcription factors, thereby modulating S-phase progression of the cell cycle, further promoting the occurrence and development of BC.

The ROC curve analysis results of this study showed that the AUC for the combined diagnosis of prognostic death in NMIBC patients using serum LAMC2 and TROAP levels was higher than that of individual diagnoses, indicating that their combined diagnosis has higher value and is of great significance for the subsequent clinical treatment of patients. LAMC2 and TROAP can serve as biomarkers for the prognostic diagnosis of NMIBC patients, and timely detection of their serum levels can help guide the clinical diagnosis and treatment of NMIBC patients.

## Conclusions

In summary, serum LAMC2 and TROAP expression is upregulated in NMIBC patients and is associated with the patients’ clinical stage and prognosis; the combined diagnosis of these two markers has significant value for the prognosis of NMIBC patients. However, this study still has certain limitations. Due to factors such as uncontrolled important confounding variables, single baseline measurements, and a relatively small sample size, the generalizability of the research results may be limited and may not fully represent a broader population. Therefore, multi-center prospective cohorts, combined with treatment information and dynamic monitoring, are needed in the future to further validate the findings of this study.
